# Discordance Between Gated Single-Photon Emission Computed Tomography Myocardial Perfusion Imaging and Coronary Angiography in Dilated Cardiomyopathy

**DOI:** 10.7759/cureus.116480

**Published:** 2026-09-18

**Authors:** Anowarul Islam, Sabrina Taher, Tanvir Ahmed, Mohammad Jewel Rana, Mahbubul Haque, Mehnaz Riffat Mou, Sadia Arju, Sabuj Biswas, Mohammad Masum Mia, Pulak Kanti Palit

**Affiliations:** 1 Cardiology, National Institute of Cardiovascular Diseases, Dhaka, BGD; 2 Radiation Oncology, Evercare Hospital Dhaka, Dhaka, BGD; 3 Radiology, Bangladesh Medical University, Dhaka, BGD

**Keywords:** coronary angiography, dilated cardiomyopathy, discordance, ischemic cardiomyopathy, myocardial perfusion imaging, nonischemic dilated cardiomyopathy

## Abstract

Background

Gated single-photon emission computed tomography myocardial perfusion imaging (SPECT-MPI) provides functional information that may help distinguish ischemic from nonischemic dilated cardiomyopathy (DCM). However, its classification may differ from that of coronary angiography (CAG). Examining these discordant cases may improve SPECT-MPI interpretation in patients with DCM. This study aimed to assess the pattern of discordance between gated SPECT-MPI and CAG in patients with DCM and describe the features of false-positive and false-negative SPECT-MPI findings.

Methodology

This cross-sectional secondary analysis included 89 patients who underwent CAG and gated SPECT-MPI during the same diagnostic sitting at the National Institute of Cardiovascular Diseases, Dhaka, Bangladesh, from October 2023 to August 2025, minimizing the possibility of intervening clinical events or therapeutic changes. We used CAG as the reference standard. We categorized patients as true-positive, true-negative, false-positive, or false-negative based on agreement between SPECT-MPI and CAG. We compared clinical, echocardiographic, angiographic, and quantitative SPECT-MPI parameters between groups.

Results

CAG classified 48 patients as ischemic cardiomyopathy and 41 as nonischemic DCM. SPECT-MPI agreed with CAG in 57 (64.0%) patients and disagreed in 32 (36.0%) patients, including 19 false-positive and 13 false-negative cases. The agreement was fair (κ = 0.269), although the association between modalities was significant (χ² = 6.545, p = 0.011). Using CAG as the reference standard, SPECT-MPI demonstrated a sensitivity of 72.9% (35/48), a specificity of 53.7% (22/41), and an overall accuracy of 64.0% (57/89). False-positive cases had higher summed stress scores, summed rest scores, summed difference scores, and transient ischemic dilation than true-negative cases. False-negative cases had lower summed stress scores, summed rest scores, and summed difference scores than true-positive cases, and most had double- or triple-vessel coronary disease.

Conclusions

In patients with DCM, gated SPECT-MPI and CAG showed discordant classification in more than one-third of cases. False-positive and false-negative patterns suggest that the two modalities provide complementary rather than fully interchangeable information. SPECT-MPI should be interpreted alongside the clinical profile, echocardiographic findings, and pre-test probability of CAD, particularly when imaging findings do not match the overall clinical picture.

## Introduction

Dilated cardiomyopathy (DCM) is a major cause of heart failure and is characterized by left ventricular dilatation and impaired systolic function. Patients with left ventricular dilatation and systolic dysfunction may present with a similar DCM phenotype despite differing underlying etiologies. A key clinical distinction is whether ventricular dysfunction is associated with significant coronary artery disease (CAD), consistent with ischemic cardiomyopathy (ICM), or occurs without significant epicardial CAD, consistent with nonischemic dilated cardiomyopathy (NIDCM) [1]. In contrast, NIDCM occurs without significant epicardial CAD and may have genetic, inflammatory, metabolic, toxic, or idiopathic causes [1-3].

In practice, differentiating ICM from NIDCM is often difficult. Both may present with exertional dyspnea, reduced left ventricular ejection fraction (LVEF), ventricular dilatation, global hypokinesia, and overlapping electrocardiographic or echocardiographic findings [4]. A history of ischemic heart disease supports an ischemic cause. Still, its absence does not exclude it, as silent myocardial infarction and unrecognized CAD may occur in patients with heart failure [5]. This distinction matters because patients with ICM may benefit from coronary revascularization in addition to guideline-directed medical therapy. In contrast, patients with NIDCM are generally managed with medical therapy, device-based treatment, or advanced heart failure care as clinically indicated [6,7].

Coronary angiography (CAG) is widely used as the reference standard for detecting obstructive epicardial CAD. However, it is invasive and provides anatomical information about the coronary lumen rather than direct assessment of myocardial perfusion, viability, scar, or microvascular dysfunction [8]. This limitation is relevant in DCM because coronary anatomy and myocardial dysfunction may not always match. In resource-limited settings, where cardiac magnetic resonance imaging or positron emission tomography (PET) may be restricted, practical noninvasive tools remain valuable.

Gated single-photon emission computed tomography myocardial perfusion imaging (SPECT-MPI) enables simultaneous evaluation of myocardial perfusion and left ventricular performance. Perfusion abnormalities may appear as fixed defects, indicating predominantly scarred myocardium, reversible defects reflecting stress-induced ischemia, or a combination of both [9]. Quantitative assessment incorporates summed stress score (SSS), summed rest score (SRS), summed difference score (SDS), transient ischemic dilation (TID), ventricular volumes, and ejection fraction to characterize perfusion abnormalities and ventricular function [10,11].

Despite these advantages, SPECT-MPI does not always agree with CAG. False-positive findings may occur because of attenuation artifacts, nonischemic fibrosis, microvascular dysfunction, conduction abnormalities, or diffuse ventricular remodeling. False-negative findings may occur with balanced ischemia, diffuse multivessel disease, or small subendocardial infarction that SPECT does not detect well [12,13]. Discordance between SPECT-MPI and CAG should therefore not be viewed only as a diagnostic error; it may also reflect the complex relationship between coronary anatomy, myocardial perfusion, scar, microvascular disease, and ventricular remodeling in DCM.

Previous nuclear perfusion imaging studies have evaluated the overall diagnostic performance of SPECT-MPI in differentiating ICM from NIDCM. Thomas et al. [14] found higher perfusion scores and more frequent reversible defects in ICM, while Singh et al. [11] reported moderate agreement between SPECT-MPI and CAG in patients with DCM. Less attention has been given to patients in whom the two modalities disagree, although this subgroup may affect diagnostic confidence, further imaging, and decisions about invasive evaluation.

Local evidence on this issue is limited. In Bangladesh and similar resource-constrained settings, understanding SPECT-MPI/CAG discordance may help clinicians interpret SPECT-MPI more cautiously and use CAG more selectively. Unlike studies focused mainly on overall diagnostic accuracy, this analysis examines false-positive and false-negative SPECT-MPI patterns in patients with DCM. The study evaluated discordance between gated SPECT-MPI and CAG and described the clinical, echocardiographic, angiographic, and quantitative SPECT-MPI characteristics of discordant cases.

## Materials and methods

Study design, setting, and duration

This cross-sectional analytical study was a secondary analysis of patients with DCM who underwent both CAG and SPECT-MPI. The study was conducted in the Departments of Cardiology and Nuclear Cardiology at the National Institute of Cardiovascular Diseases (NICVD), Dhaka, Bangladesh, from October 2023 to August 2025.

Study population and sampling technique

The study population consisted of patients with DCM evaluated at NICVD during the study period who underwent both CAG and gated SPECT-MPI. Participants were enrolled using purposive sampling. For this secondary analysis, we included all eligible patients with complete clinical, echocardiographic, CAG, and gated SPECT-MPI data.

Inclusion criteria

Patients were eligible for inclusion if they were aged 18 years or older. They had clinical and echocardiographic features of DCM, including exertional breathlessness with New York Heart Association (NYHA) functional class II or III symptoms, LVEF <40%, global left ventricular hypokinesia, and left ventricular dilatation on echocardiography. For the purposes of this study, the term DCM was used operationally to describe the presenting phenotype of left ventricular dilatation with global systolic dysfunction before etiological classification. Subsequent coronary angiography was used to distinguish patients with significant epicardial CAD, classified as ICM, from those without significant CAD, classified as NIDCM.

Exclusion criteria

Patients were excluded if they had acute coronary syndrome; previous percutaneous coronary intervention (PCI) or coronary artery bypass grafting (CABG); an implanted cardiac device; LVEF <20%; NYHA functional class I or IV symptoms; cardiac arrhythmia; electrocardiogram (ECG) evidence of previous myocardial infarction; significant valvular or congenital heart disease; chronic kidney disease; pregnancy or lactation; or age <18 years. Patients who underwent coronary revascularization or experienced a major cardiovascular event between CAG and gated SPECT-MPI were also excluded.

Sample size and analytic population

The original sample size was determined for the parent study using the Buderer formula [15] for diagnostic accuracy studies. The calculation incorporated an expected specificity of 66.67% for myocardial perfusion imaging, based on Singh et al. [11], an estimated DCM prevalence of 0.40% in the source population, a 95% confidence level, and an absolute precision of 0.10. The present study represents a secondary analysis of that parent cohort; therefore, no separate sample size calculation was performed for the current discordance analysis. All 89 eligible participants with complete paired CAG and gated SPECT-MPI data were included.

Clinical and echocardiographic assessment

We recorded baseline demographic, clinical, ECG, and echocardiographic information using a structured data collection sheet. We performed echocardiography using a Philips EPIQ 7C system. LVEF and left ventricular end-diastolic volume (LVEDV) were measured using the biplane Simpson method, while left ventricular internal diameter in diastole (LVIDd) was measured from standard echocardiographic views.

Coronary angiography

All patients underwent CAG through radial or femoral arterial access. Experienced cardiologists interpreted angiograms and were blinded to gated SPECT-MPI findings. Significant CAD was defined as ≥70% luminal stenosis in at least one major epicardial coronary artery or ≥50% stenosis of the left main coronary artery. We classified patients meeting these criteria as having ICM and those without significant angiographic CAD as having NIDCM. We used CAG as the reference standard.

Gated single-photon emission computed tomography myocardial perfusion imaging

Gated SPECT-MPI was performed within a predefined interval after CAG using a standard stress-rest protocol. Patients fasted overnight, avoided caffeine for at least 12 hours, and withheld beta-blockers and nitrates for 24 hours when clinically feasible. We performed exercise stress testing using the Bruce treadmill protocol until the target heart rate was achieved or standard termination criteria were met. At peak stress, we administered 10 millicuries (mCi) of technetium-99m sestamibi, followed by stress imaging. Rest imaging was performed after administering 20 mCi of technetium-99m sestamibi.

Images were acquired using a CorCam™ dedicated dual-head cardiac SPECT gamma camera (DDD-Diagnostic A/S, Hørsholm, Denmark) over a 180° arc and reconstructed using Oasis software. We performed quantitative analysis using Corridor4DM™ software (INVIA Medical Imaging Solutions, Ann Arbor, MI, USA). An experienced nuclear imaging physician, blinded to the CAG findings, evaluated perfusion defect distribution, severity, and reversibility. Quantitative parameters included SSS, SRS, SDS, TID, ventricular volumes, and gated LVEF.

Gated SPECT-MPI was classified as suggestive of ICM when perfusion defects involved two or more contiguous myocardial segments on stress or rest imaging, or when any reversible perfusion defect was present. We reviewed all studies for motion, attenuation artifacts, and overall image quality before final interpretation.

Potential confounders

Potential demographic and cardiovascular confounding variables considered in the analysis included age, sex, hypertension, diabetes mellitus, smoking status, dyslipidemia, family history of CAD, LVEF, LVIDd, and indexed LVEDV. We compared these variables between relevant diagnostic groups to assess whether differences in clinical or ventricular characteristics could contribute to the observed discordance between gated SPECT-MPI and CAG. We did not perform multivariable regression analysis because of the limited number of false-positive and false-negative cases; therefore, we could not completely exclude residual confounding.

Definition of concordance and discordance

Patients were categorized according to agreement between gated SPECT-MPI and CAG. Concordant cases comprised true-positive and true-negative findings. Discordant cases comprised false-positive findings, in which gated SPECT-MPI suggested ICM despite CAG showing NIDCM, and false-negative findings, in which gated SPECT-MPI suggested NIDCM despite CAG demonstrating significant CAD consistent with ICM.

Statistical analysis

We analyzed data using SPSS Statistics for Windows, Version 25.0 (IBM Corp., Armonk, USA). We summarized categorical variables as frequencies and percentages. Continuous variables were expressed as mean ± standard deviation (SD) or median with interquartile range, depending on their distribution. Categorical variables were compared using the chi-square test or Fisher’s exact test, as appropriate. Continuous variables were compared using the independent-samples Student’s t-test or Mann-Whitney U test, depending on data distribution. We assessed agreement between gated SPECT-MPI and CAG using Cohen’s kappa coefficient. We calculated diagnostic performance measures, including sensitivity, specificity, positive predictive value, negative predictive value, and overall accuracy, using CAG as the reference standard. Analyses were performed using available data. A two-sided p-value <0.05 was considered statistically significant.

Ethical considerations

The parent study was approved by the Ethical Review Committee of the NICVD, Dhaka, Bangladesh (approval number: NICVD/Academic/Ethical/2023/65; October 5, 2023). The present secondary analysis used anonymized data collected under the approved study protocol and involved no additional patient contact or intervention. We obtained written informed consent from all participants before enrollment. The study followed the Declaration of Helsinki, and participant confidentiality was maintained throughout.

## Results

A total of 89 patients with DCM were analyzed. CAG classified 48 patients as ICM and 41 as NIDCM. By gated SPECT-MPI, 54 patients were classified as ICM and 35 as NIDCM. SPECT-MPI and CAG were concordant in 57 (64.0%) patients and discordant in 32 (36.0%) patients. The concordant group included 35 true-positive and 22 true-negative cases. The discordant group included 19 false-positive and 13 false-negative SPECT-MPI findings. The association between the two modalities was statistically significant (χ² = 6.545, p = 0.011), but agreement was only fair (κ = 0.269). Using CAG as the reference standard, SPECT-MPI showed a sensitivity of 72.9% (35/48), specificity of 53.7% (22/41), positive predictive value of 64.8% (35/54), negative predictive value of 62.9% (22/35), and overall accuracy of 64.0% (57/89), as shown in Table 1.

**Table 1:** Agreement and diagnostic performance of gated SPECT-MPI using CAG as the reference standard. Data are presented as numbers (n) or percentages, as applicable. The association between SPECT-MPI and CAG classifications was assessed using the Pearson chi-square test, while agreement was evaluated using Cohen’s kappa coefficient. A two-sided p-value <0.05 was considered statistically significant. CAG = coronary angiography; ICM = ischemic cardiomyopathy; NIDCM = nonischemic dilated cardiomyopathy; SPECT-MPI = single-photon emission computed tomography myocardial perfusion imaging

Parameter	Value
Ischemic cardiomyopathy (ICM) by SPECT-MPI/ICM by CAG	35
ICM by SPECT-MPI/NIDCM by CAG	19
NIDCM by SPECT-MPI/ICM by CAG	13
NIDCM by SPECT-MPI/NIDCM by CAG	22
ICM by CAG, total	48
NIDCM by CAG, total	41
ICM by SPECT-MPI, total	54
NIDCM by SPECT-MPI, total	35
Total patients	89
Sensitivity, n/N (%)	35/48 (72.9%)
Specificity, n/N (%)	22/41 (53.7%)
Positive predictive value, n/N (%)	35/54 (64.8%)
Negative predictive value, n/N (%)	22/35 (62.9%)
Overall accuracy, n/N (%)	57/89 (64.0%)
Pearson χ²	6.545
P-value	0.011
Cohen’s κ	0.269

Clinical and echocardiographic profiles were largely similar between concordant and discordant cases. Median age was 55 years in both groups. Hypertension, diabetes, smoking, dyslipidemia, family history of CAD, LVEF, LVIDd, and LVEDV index did not differ significantly. Male sex was less frequent in discordant cases than in concordant cases, but the difference was borderline and not statistically significant: 15/32 (46.9%) versus 39/57 (68.4%) (p = 0.070).

False-positive SPECT-MPI cases showed greater perfusion abnormalities than true-negative cases, with higher SSS (21.00 vs. 10.50, p < 0.001), SRS (22.00 vs. 8.50, p < 0.001), SDS (4.00 vs. 1.00, p = 0.015), and TID (0.98 vs. 0.74, p < 0.001). Small but statistically significant differences were also observed in LVEF (35.00% vs. 34.00%, p = 0.045), LVIDd (59.00 vs. 61.50 mm, p = 0.049), and indexed LVEDV (108.05 vs. 118.22 mL/m², p = 0.038). Most false-positive cases had fixed perfusion defects, followed by reversible and mixed defects, as shown in Table 2.

**Table 2:** Comparison of false-positive and true-negative SPECT-MPI findings. Continuous variables are presented as medians (IQR) and were compared using the Mann-Whitney U test. Perfusion-defect patterns are presented as counts (n) and were analyzed descriptively. A two-sided p-value <0.05 was considered statistically significant. LVEF is expressed as %, LVIDd as mm, and LVEDV index as mL/m². SSS, SRS, SDS, and TID are dimensionless indices. IQR = interquartile range; LVEDV = left ventricular end-diastolic volume; LVEF = left ventricular ejection fraction; LVIDd = left ventricular internal diameter in diastole; SDS = summed difference score; SPECT-MPI = single-photon emission computed tomography myocardial perfusion imaging; SRS = summed rest score; SSS = summed stress score; TID = transient ischemic dilation

Variable	False-positive, n = 19	True-negative, n = 22	P-value
SSS, median (IQR)	21.00 (15.50–30.00)	10.50 (7.25–13.75)	<0.001
SRS, median (IQR)	22.00 (10.50–27.50)	8.50 (7.00–12.00)	<0.001
SDS, median (IQR)	4.00 (1.50–7.50)	1.00 (0.00–2.75)	0.015
TID, median (IQR)	0.98 (0.91–1.03)	0.74 (0.72–0.88)	<0.001
LVEF, %, median (IQR)	35.00 (33.50–38.00)	34.00 (31.25–35.00)	0.045
LVIDd, mm, median (IQR)	59.00 (57.00–62.50)	61.50 (59.00–65.75)	0.049
LVEDV index, mL/m², median (IQR)	108.05 (99.22–118.43)	118.22 (109.77–146.38)	0.038
Fixed defect, n	14	0	—
Reversible defect, n	4	0	—
Fixed and reversible defect, n	1	0	—
No perfusion defect, n	0	22	—

False-negative cases had lower perfusion scores than true-positive cases despite having angiographically significant CAD. SSS (15.00 vs. 30.00, p < 0.001), SRS (12.00 vs. 23.00, p = 0.004), and SDS (2.00 vs. 5.00, p = 0.010) were all significantly lower in false-negative cases. TID was also lower but did not reach statistical significance (0.90 vs. 1.03, p = 0.082). Male sex was significantly less common in false-negative than true-positive cases, 5/13 (38.5%) vs 30/35 (85.7%), p = 0.002, as shown in Table 3. Moreover, false-negative findings were not limited to mild coronary disease. Among the 13 false-negative cases, eight had double-vessel disease, four had triple-vessel disease, and one had single-vessel disease. This indicates that SPECT-MPI discordance occurred even in patients with multivessel coronary artery involvement (Table 3).

**Table 3:** Comparison of false-negative and true-positive SPECT-MPI findings. Sex distribution is presented as numbers and percentages, n (%), and was compared using Fisher’s exact test. Continuous variables are presented as median (IQR) and were compared using the Mann-Whitney U test. Coronary vessel involvement is presented as counts (n) and was analyzed descriptively. A two-sided p-value <0.05 was considered statistically significant. LVEF is expressed as %, LVIDd as mm, and LVEDV index as mL/m². SSS, SRS, SDS, and TID are dimensionless indices. IQR = interquartile range; LVEDV = left ventricular end-diastolic volume; LVEF = left ventricular ejection fraction; LVIDd = left ventricular internal diameter in diastole; SDS = summed difference score; SRS = summed rest score; SSS = summed stress score; TID = transient ischemic dilation

Variable	False-negative, n = 13	True-positive, n = 35	P-value
Male sex, n (%)	5 (38.5)	30 (85.7)	0.002
SSS, median (IQR)	15.00 (12.00–18.00)	30.00 (25.50–34.50)	<0.001
SRS, median (IQR)	12.00 (11.00–17.00)	23.00 (15.00–28.50)	0.004
SDS, median (IQR)	2.00 (1.00–3.00)	5.00 (2.50–10.00)	0.010
TID, median (IQR)	0.90 (0.83–0.93)	1.03 (0.91–1.13)	0.082
LVEF, %, median (IQR)	35.00 (34.00–37.00)	33.00 (32.00–37.00)	0.440
LVIDd, mm, median (IQR)	62.00 (58.00–66.00)	60.00 (59.00–64.00)	0.770
LVEDV index, mL/m², median (IQR)	119.01 (109.61–132.96)	116.29 (104.55–125.81)	0.531
Single-vessel disease, n	1	1	—
Double-vessel disease, n	8	19	—
Triple-vessel disease, n	4	15	—

Figure 1 shows the distribution of SSS across true-positive, false-positive, false-negative, and true-negative groups.

**Figure 1:**
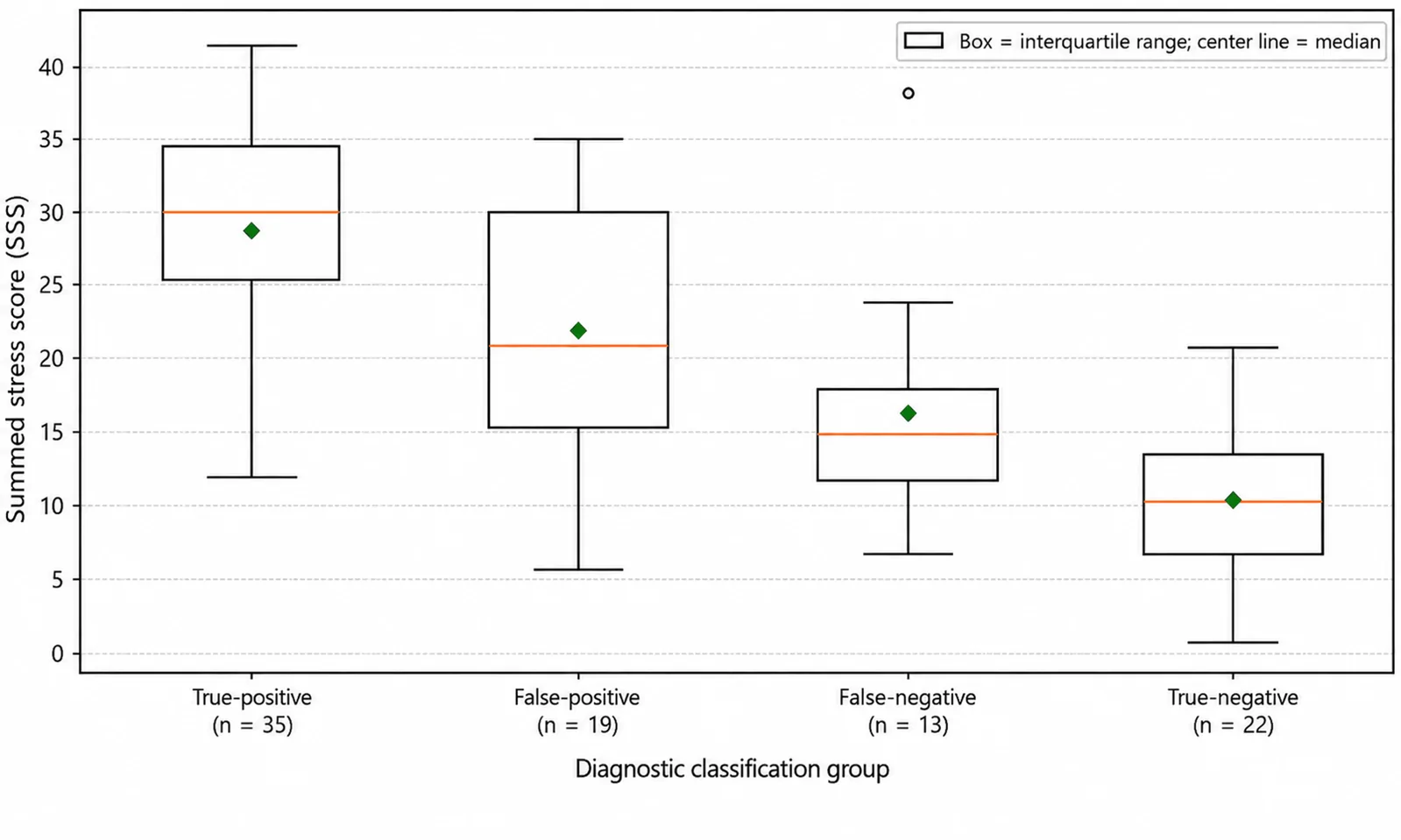
Distribution of SSS across SPECT-MPI/CAG diagnostic groups. Box-and-whisker plot of SSS among true-positive, false-positive, false-negative, and true-negative SPECT-MPI classifications, using CAG as the reference standard. Boxes represent the IQR with the median line; whiskers indicate 1.5× the IQR; circles indicate outliers, and the diamond represents the mean. CAG = coronary angiography; IQR = interquartile range; SPECT-MPI = single-photon emission computed tomography myocardial perfusion imaging; SSS = summed stress score

## Discussion

This study examined concordant and discordant etiological classifications by gated SPECT-MPI and CAG in patients with DCM. Although the two modalities were significantly associated, agreement was only fair, supporting the central finding that SPECT-MPI and CAG provide related but not interchangeable information when distinguishing ICM from NIDCM.

This discordance is clinically plausible because the tests assess different aspects of the disease. CAG identifies obstructive epicardial coronary stenosis, whereas gated SPECT-MPI reflects myocardial perfusion, scar burden, inducible ischemia, ventricular volumes, and systolic function [9-11]. In DCM, coronary anatomy may not align directly with perfusion or ventricular dysfunction. CAG also does not assess microvascular disease, myocardial fibrosis, viability, or tissue characteristics [8,16]. Thus, disagreement between SPECT-MPI and CAG may reflect both technical factors and the heterogeneous myocardial substrate of DCM.

We observed false-positive SPECT-MPI findings in 19 patients classified as ICM by SPECT-MPI despite the absence of angiographically significant CAD. Compared with true-negative cases, false-positive cases had higher SSS, SRS, SDS, and TID, and most showed fixed perfusion defects. They also showed modest differences in LVEF, LVIDd, and indexed LVEDV. These structural and functional differences were modest and should be interpreted cautiously given the small subgroup size and multiple comparisons. This suggests that perfusion defects may occur in DCM even without obstructive epicardial CAD. Possible explanations include nonischemic fibrosis, microvascular dysfunction, attenuation artifact, patchy tracer uptake, conduction-related defects, or altered count distribution in dilated ventricles [12,16,17]. Fixed defects are often interpreted as ischemic scars, but in NIDCM, they may also reflect nonischemic fibrosis or other myocardial injury. Without tissue characterization, SPECT-MPI cannot reliably separate ischemic scar from nonischemic fibrosis; cardiac magnetic resonance is better suited for this distinction, although it may not be widely available [8,18,19].

False-negative SPECT-MPI findings were observed in 13 patients with angiographically significant CAD who were classified as NIDCM. Compared with true-positive cases, they had lower SSS, SRS, and SDS, while TID was also lower but not statistically significant. This pattern may occur when significant coronary stenosis does not produce a clear regional perfusion defect on SPECT-MPI. Balanced ischemia, diffuse multivessel disease, small subendocardial infarction, limited spatial resolution, and globally reduced tracer uptake in a remodeled left ventricle may contribute [12,13]. Importantly, most false-negative cases had double-vessel or triple-vessel disease, indicating that a nonischemic SPECT-MPI pattern does not completely exclude significant CAD in patients with DCM.

Previous nuclear perfusion imaging studies using both SPECT and PET have demonstrated differences between ischemic and nonischemic cardiomyopathy. Singh et al. [11] reported moderate agreement between SPECT-MPI and CAG, while Majmudar et al. [20], using PET, demonstrated substantially greater perfusion abnormality in ICM than NIDCM. Vavlukis et al. [21] found that fixed and reversible defects favored ICM, whereas patchy uptake and left bundle branch block were more common in NIDCM. This analysis adds to this evidence by focusing on discordant cases rather than only sensitivity, specificity, and accuracy.

These findings are relevant for Bangladesh and similar resource-limited settings, where SPECT-MPI may be more accessible than cardiac magnetic resonance or PET. Previous local experience supports using gated SPECT-MPI to assess perfusion defects and ventricular function in patients with dilated ventricles [22]. However, these results emphasize that SPECT-MPI should be interpreted alongside clinical findings, ECG, echocardiography, and pre-test probability of CAD. This integrated approach may reduce overdiagnosis of ICM in false-positive cases and under-recognition of CAD in false-negative cases.

This study has several limitations. It was a single-center secondary analysis with a modest sample size, particularly in the false-positive and false-negative subgroups, which limited statistical power and generalizability. Purposive sampling may have introduced selection bias. Because we did not perform multivariable regression, residual confounding may remain. We used CAG as the reference standard, although it does not assess microvascular dysfunction, fibrosis, viability, or tissue characteristics. Advanced imaging was unavailable, preventing confirmation of the mechanisms of discordance. Finally, the cross-sectional design limited prognostic assessment.

Overall, SPECT-MPI/CAG discordance was not uncommon in this DCM cohort and has practical implications for interpretation. False-positive cases showed higher perfusion abnormality scores despite no significant angiographic CAD, whereas false-negative cases had lower perfusion scores despite significant coronary disease, often with multivessel involvement. These findings support using SPECT-MPI as part of an integrated diagnostic assessment rather than as a standalone substitute for angiographic or clinical judgment.

## Conclusions

In patients with dilated left ventricular systolic dysfunction, gated SPECT-MPI and CAG were discordant in approximately one-third of cases. A negative or nonischemic SPECT-MPI pattern should not, by itself, exclude obstructive CAD when the clinical likelihood remains high, particularly in patients with multivessel disease. Conversely, abnormal SPECT-MPI findings in the absence of obstructive epicardial CAD should prompt review for technical artifacts and, where clinically appropriate and available, consideration of cardiac magnetic resonance tissue characterization or evaluation for microvascular dysfunction. Decisions regarding additional anatomical imaging or invasive coronary angiography should be guided by symptoms, clinical likelihood of CAD, ventricular function, and overall risk rather than SPECT-MPI alone. The subgroup observations in this study are exploratory and require confirmation in larger prospective cohorts.
